# Importance of pH in Synthesis of pH-Responsive Cationic Nano- and Microgels

**DOI:** 10.3390/polym13050827

**Published:** 2021-03-08

**Authors:** Marco Annegarn, Maxim Dirksen, Thomas Hellweg

**Affiliations:** Department of Physical and Biophysical Chemistry, Bielefeld University, Universitätsstraße 25, 33615 Bielefeld, Germany; m.annegarn@uni-bielefeld.de (M.A.); dirksen@uni-bielefeld.de (M.D.)

**Keywords:** microgels, cationic, pH, acrylamides

## Abstract

While cationic microgels are potentially useful for the transfection or transformation of cells, their synthesis has certain drawbacks regarding size, polydispersity, yield, and incorporation of the cationic comonomers. In this work, a range of poly(*N*-isopropylacrylamide) (PNIPAM) microgels with different amounts of the primary amine *N*-(3-aminopropyl)methacrylamide hydrochloride (APMH) as the cationic comonomer were synthesized. Moreover, the pH-value during reaction was varied for the synthesis of microgels with 10 mol% APMH-feed. The microgels were analyzed by means of their size, thermoresponsive swelling behavior, synthesis yield, polydispersity and APMH-incorporation. The copolymerization of APMH leads to a strong decrease in size and yield of the microgels, while less than one third of the nominal APMH monomer feed is incorporated into the microgels. With an increase of the reaction pH up to 9.5, the negative effects of APMH copolymerization were significantly reduced. Above this pH, synthesis was not feasible due to aggregation. The results show that the reaction pH has a strong influence on the synthesis of pH-responsive cationic microgels and therefore it can be used to tailor the microgel properties.

## 1. Introduction

Nano- and microgels are crosslinked gel-particles with colloidal dimensions that emerged as a research field in polymer sciences in recent decades [1,2,3,4,5,6,7,8]. Their increasing popularity can be attributed to their responsiveness towards external stimuli such as temperature or pH, which is achieved by polymerizing certain monomers such as acrylamides and weak acids or bases [9,10,11,12,13,14] and due to their tunability in size [15] and architecture [16,17,18,19]. A rapid change in size upon changing these stimuli enables a vast variety of possible applications in the fields of catalysis, sensing, drug targeting, surface-functionalization or bio-conjugation [7,20,21,22,23,24,25,26,27,28,29,30,31]. The latter is often mentioned together with cationic nano- and microgels that are far less studied than their anionic counterparts [25].

Cationic polymers can be conjugated with biomolecules such as peptides, antibodies, and nucleic acids for different purposes [32,33,34,35,36,37,38]. Especially the replacement of controversial viral vectors with cationic polymers for delivery of nucleic acid contructs experiences emerging interest in the context of genetic therapies or vaccination [39]. Polymers such as polyethyleneimine (PEI) in its linear or branched form are already in use for the transfection of cells [32,40]. Since a lower efficiency and higher cytotoxicity of polymer-based vectors are still an issue, further developments are required [40]. Possible benefits regarding a straight-forward synthesis, easily adjustable properties and their responsiveness highlight cationic microgels as possible non-viral vectors. Additionally, the given structure of nano- and microgels seems perfect for the use as transfection-agents: while nucleic acids only adsorb at the outermost cationic groups, inner, sterically hindered, charged moieties are able to facilitate the necessary disruption of endosomes after endocytosis through protonation (proton sponge effect) [41].

However, to reversibly immobilize nucleic acids or peptides with nano- or microgels, a sufficient amount of cationic groups (e.g., stemming from amines) has to be incorporated into their network [40]. To achieve this, various approaches including the post-synthetic introduction of cationic charges (e.g., hydrolysis of polyvinylformamide to polyvinylamine or “click” chemistry), addition of salts to screen charges, synthesis in reverse micelles or the use of anionic radical initiators have been published [12,36,41,42,43,44,45]. However, these solutions can have certain drawbacks, as they render the synthesis more complex, could induce aggregation and aspherical deformations or introduce (possibly unwanted) anionic charges into the microgels.

Using the well-established method of precipitation polymerization, different issues arise at elevated concentrations of the cationic monomers. For various polymer systems, a decreasing microgel size along with high dispersities, poor synthesis yields and low incorporation efficiencies of the cationic comonomer are reported consistently for increased concentrations of the comonomer [12,36,46,47,48]. A work by Meunier et al. on cationic microgels based on *N*-isopropylacrylamide (NIPAM) and the amine 2-aminoethylmethacrylate hydrochloride (AEMH) reveals a clear influence of the cationic comonomer on size, yield and polydispersity of the obtained cationic microgels even at minor AEMH-feeds below 5 mol% [47].

A detailed study by Mai-ngam et al. on the polymerization kinetics of NIPAM and the primary amine *N*-(3-aminopropyl)methacrylamide hydrochloride (APMH) to linear copolymers highlights the origin of these problems [42]. Due to reactivity ratios below one, both NIPAM and APMH are located rather randomly along the polymer chains. The Coulombic repulsion and enhanced hydrophilicity, caused by the cationic charges, are shifting the lower critical solution temperature (LCST) of the polymer chains to higher values, impeding or even preventing a chain-collapse at ordinary synthesis temperatures [42]. This collapse of the polymer chains is crucial for the generation of microgels by precipitation polymerization, as it is the first necessary step in particle formation and consecutive growth. Instead, a large number of water-soluble oligomers and linear polymers are generated during polymerization that do not substantially contribute to particle formation and can be considered to be unwanted byproducts [12,36,47]. Additionally, a stabilizing, surfactant-like nature might be attributed to the charged water-soluble polymers, resulting in a further decrease of the microgel size [49].

Since cationic microgels frequently bear pH-responsive moieties such as amines, an adjustment of the reaction pH in order to eliminate the cationic charges could serve as an easy and effective solution for the problems associated with the incorporation of the cationic comonomer. When performing the synthesis at an altered pH, with the cationic monomer being mostly uncharged, the suppression of the chain-collapse should be reduced to a minimum. For the primary amine APMH ($pK_{a}\approx 10$) [36], a significant reduction of the known problems can be expected for reaction pH above 8–9. The influence of the pH-value on cationic or anionic microgels after synthesis and purification is investigated quite frequently [11,12,47]. An adjustment of the reaction pH is rather uncommon in microgel synthesis, though a strong influence of the pH on reaction kinetics of (de)protonateable monomers is well known [50,51,52]. A study by Zha et al. on the influence of both dimethylaminoethyl methacrylate (DMAEMA) concentration and reaction pH on poly(NIPAM-*co*-DMAEMA) microgels, found a high impact of pH on the microgel size [53]. A recent study by Karanastasis et al. on anionic microgels from NIPAM and methacrylic acid (MAA) reveals a strong influence of the reaction pH on the microgel size and MAA distribution [54]. A change in reactivity upon deprotonation of MAA and a surfactant-like character of charged oligomers at high pH are given as the main explanations.

The intention of this work is to determine the impact of the cationic comonomer APMH on the synthesis of poly(NIPAM-*co*-APMH) microgels and the influence of the reaction pH on the synthesis of these amine-bearing cationic microgels. We aim at understanding the interplay between APMH content, particle size and dispersity. This will allow tailoring cationic microgels e.g., for nucleic acid complexation.

## 2. Material and Methods

### 2.1. Chemicals

If not stated otherwise, all chemicals were used without further purification. For microgel syntheses *N*-isopropylacrylamide (NIPAM, 97%; TCI Germany GmbH, Eschborn, Germany) recrystallized from *n*-hexane (p.a.; VWR International GmbH, Darmstadt, Germany), *N*-(3-aminopropyl)methacrylamide hydrochloride (APMH, 97%; Sigma Aldrich, Munich, Germany), *N*,*N*${}^{\prime }$-methylenebisacrylamide (BIS, 99%; Sigma Aldrich) and 2,2${}^{\prime }$-azobis(2-methylpropionamidine) dihydrochloride (V50, 97% granular; Sigma Aldrich) were used. Deuteriumoxide (99.9%) and deuteriumchloride solution (36–38% in D_2_O) were purchased from Deutero GmbH, Kastellaun, Germany. Sodium hydroxide solution and hydrochloric acid (0.1 M, Fisher Chemical, Waltham, MA, USA) were used for pH-adjustments, sodium chloride (>99.9%; Carl Roth, Karlsruhe, Germany) was used to alter ionic strength. Water was purified using an Arium pro VF system (Sartorius AG, Göttingen, Germany).

### 2.2. Synthesis and Purification of Microgels

All microgel syntheses were carried out as surfactant-free precipitation polymerizations in a three neck round bottom flask (250 mL) under nitrogen atmosphere, equipped with a reflux condenser with gas bubbler, mechanical stirrer (RZR2021, Heidolph Instruments, Schwabach, Germany) and N_2_-inlet. NIPAM (11.55 mmol), BIS (0.58 mmol, 5 mol%) and APMH (0–1.16 mmol, 0–10 mol%) were dissolved in water (149 mL). The reaction mixture was heated to 80 ${}^{\circ }C$ oil-bath temperature and simultaneously nitrogen was bubbled through the solution under continuous stirring (400 rpm). After reaching the target temperature, the solution pH was measured (Lab 860 pH-meter, gel electrode, Schott Instruments, now SI Analytics GmbH, Mainz, Germany) until a constant value was obtained. For certain syntheses, the pH was adjusted by addition of HCl/NaOH ( 0.1 M). After a total equilibration time of one hour, the reaction was initiated by the addition of freshly prepared V50-solution (1 mL, 0.404 M), followed by a visible increase in turbidity. After 4 h reaction time, the oil-bath was removed and the reaction solution was stirred overnight without N_2_-atmosphere at room temperature.

The purification of microgels was achieved by five cycles of centrifugation (Optima L-90K, Beckman Coulter GmbH, Krefeld, Germany) of the suspensions, removal of the supernatant and re-dispersing the microgels in water. Centrifugation speed and duration were adjusted individually for every microgel and cycle to the respective sedimentation properties. For nanogels and smaller microgels ($R_{h}<100\;nm$) purification was performed by concentrating the suspensions by single ultracentrifugation and subsequent dialysis against water for several days with multiple water changes, until conductivity of the dialysate remained constant. The total mass of microgel in the purified dispersion and the amount of water-soluble polymer (WSP) in the first supernatant were determined gravimetrically (triple determination) from aliquots.

### 2.3. Photon Correlation Spectroscopy (PCS)

Measurements were performed on microgel dispersions, highly diluted with a low-ionic-strength (6 mM) phosphate buffer at pH = 7.4. All solutions were prepared under dust-free conditions.

#### 2.3.1. Angle-Dependent PCS

Angle-dependent PCS measurements were carried out on a 3D LS Spectrometer Pro (LS Instruments AG, Fribourg, Switzerland) in auto-mode at 10 ${}^{\circ }C$. The setup with a dual beam 3D scattering-geometry consists of a HeNe-LASER (JDSU 1145P, 632.8 nm; Thorlabs Inc., Newton, NJ, USA), thermostatically controlled decaline index-matching bath and automated goniometer with two detectors (SPCM-AQRH-13-FC, Perkin Elmer, Waltham, MA, USA). The samples were measured between 35${}^{\circ }$ and 120${}^{\circ }$ in increments of 5${}^{\circ }$. At each angle, three measurements with 120 s duration were performed. The obtained data was evaluated using a second order cumulant fit [55,56]. With the dependence of the measured decay rate Γ on the squared magnitude of the scattering vector $q^{2}$ given in Equation (1), the z-averaged translational diffusion coefficient $D_{T}$ was obtained by linear regression.

(1) $$\Gamma =D_{T}\;\cdot \;q^{2}$$

The magnitude *q* of the scattering vector depends on the laser wavelength λ, refractive index *n* and the scattering angle θ.

(2) $$q=\frac{4\pi n}{\lambda }\sin\left(\frac{\theta }{2}\right)$$

From the translational diffusion coefficient, hydrodynamic radii $R_{h}$ were calculated via Stokes-Einstein relation (Equation (3)) for spherical particles - with Boltzmann-constant $k_{B}$, solvent viscosity η and absolute Temperature *T*.

(3) $$R_{h}=\frac{k_{B}T}{6\pi \eta D_{T}}$$

The viscosity of the buffer was assumed to be equal to water and was calculated depending on sample temperature [57].

#### 2.3.2. Temperature-Dependent PCS

Swelling curves were measured with a self-built PCS setup, consisting of a HeNe-LASER (HNL210L, 632.8 nm; Thorlabs Inc.), multiple-τ correlator (ALV-6010, ALV-Laser Vertriebsgesellschaft m-b.H., Langen, Germany), thermostatically controlled decaline index-matching-bath and single photon detector (ALV/SO-SIPD; ALV-Laser Vertriebsgesellschaft m-b.H.). Five measurements of 200 s each per temperature (10–60 ${}^{\circ }C$) at a scattering-angle of 45${}^{\circ }$ in pseudo-cross-correlation-mode were performed. After every temperature change, the sample was equilibrated for 25 min. The obtained data was evaluated using a second order cumulant fit. Hydrodynamic radii were calculated according to Equations (1)–(3) for the single value of *q* corresponding to $\theta =45{}^{\circ }$.

### 2.4. Atomic Force Microscopy (AFM)

Samples were prepared by pipetting a diluted dispersion of the microgel on silicon wafers (Siegert Wafer GmbH, Aachen, Germany) that were freshly cleaned with oxygen-plasma (Zepto, 0,4–0,6 m O_2_, 100%, 60 s; Diener Electronics, Ebhausen, Germany). After waiting (10 min), the remaining liquid was removed by spinning the wafer on a spin-coater (LabSpin6, SÜSS MicroTec, Garching, Germany, 1000–1500 rpm, 300 s). Atomic force microscopy was performed in tapping mode (amplitude-modulation) with a Nanoscope IIIa (Digital Instruments, now Bruker Corp., Billerica, MA, USA) mounted on an inverse optical microscope (Zeiss Axiovert135, Carl Zeiss Microscopy GmbH, Oberkochen, Germany) or with a Multimode V with Nanoscope V controller (Bruker Corp., Karlsruhe, Germany). TAP300Al-G (*k* = 40 N $m^{-1}$, $\nu_{e}$ = 300 kHz; Budget Sensors, Innovative Solution Bulgaria Ltd., Sofia, Bulgaria) were used as cantilevers. Images were leveled, corrected and analyzed with open-source software Gwyddion [58].

### 2.5. Nuclear Magnetic Resonace Spectroscopy

Samples containing 5 mg microgel and 0.1 M DCl were prepared by diluting aqueous microgel dispersions with D_2_O and concentrated DCl and were transferred into NMR tubes (Boroeco-5-7, Deutero GmbH, Kastellaun, Germany). ${}^{1}H-\mathrm{NMR}$ spectra were measured on an Avance III 500 (500 MHz) (Bruker Corp.) with solvent peak suppression. Spectra were corrected for phase and baseline (Whittaker Smoother) prior to integration of signals.

## 3. Results and Discussion

### 3.1. Variation of APMH-Feed

Poly(NIPAM-*co*-APMH) microgels with nominally 0.0, 2.5, 5.0 and 10.0 mol% APMH-feed were obtained by standard precipitation polymerization and subsequent purification. The reaction pH was measured prior to initiation and was found to be slightly basic (7.6) for synthesis without APMH and very acidic (3.3, 3.1 and 2.6) with increasing APMH-feed (see Table 1). While the reaction solution without APMH turned completely opaque after initiation, increasing amounts of APMH lowered the turbidity. With 10.0 mol% APMH only a small change in turbidity was visible after initiation. The pH-shift by addition of APMH can not be explained by the APMH acting as a weak acid in its protonated state. Most likely the acidic pH is caused by excess hydrochloric acid, as APMH is a hydrochloride. Since the monomeric form of APMH has a p$K_{a}$-value of approximately 10, APMH is copolymerized in its protonated and therefore cationic (R−NH_3_^+^) state.

As shown in Table 1, the microgel yield decreases continuously from 54% to 24% with increasing APMH-feed. Likewise, the amount of water soluble polymer (WSP) in the supernatant after first centrifugation increases from 32% to 73%. This halving of microgel yield by addition of 10 mol% APMH, was also found by Hu et al. for poly(NIPMAM-*co*-APMH) microgels with 9 mol% APMH [12]. Because of the stabilization of the polymer chains by cationic charges, the LCST is shifted to higher temperatures, impeding or even preventing the collapse of APMH-rich polymer chains [36,42]. As these stabilized chains are unlikely to contribute to the formation and growth of microgel particles, they are consequently removed as WSP during purification and have to be considered to be unwanted byproducts, lowering the overall yield.

**Figure 1:**
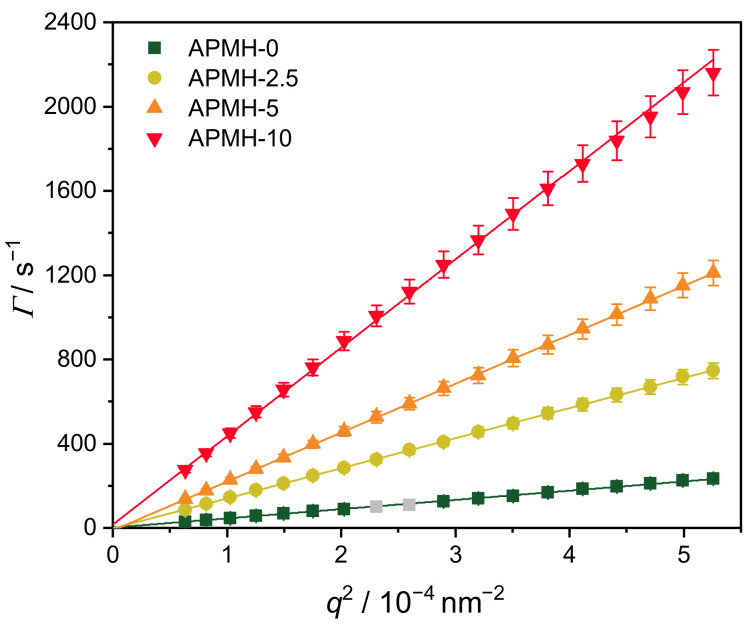
Relaxation rates Γ measured by angle-dependent PCS plotted against $q^{2}$ with linear regressions. Grey data points were excluded due to deviations resulting from a formfactor minimum.

The hydrodynamic radius and dispersity of the synthesized microgels were analyzed by angle-dependent PCS measurements at 10 ${}^{\circ }C$. The obtained relaxation rates Γ are plotted vs. $q^{2}$ in Figure 1. The expected linear dependence according to Equation (1) is found. Within the experimental precision the intercept in Figure 1 is zero. Hence, only center of mass diffusion of the microgel particles is observed in our experiments. Only small deviations are visible, for example caused by a local formfactor minimum of the largest microgel APMH-0. The hydrodynamic radii $R_{h}$ were calculated from the slopes according to Equation (3). Both hydrodynamic radii and dispersities are listed in Table 1. The addition of only 10 mol% APMH leads to a reduction in hydrodynamic radius from (361±18) nm (APMH-0) down to (37.7±1.9) nm (APMH-10) due to the increased formation of WSPs. As these do not contribute to microgel growth, smaller microgels are obtained. According to IUPAC gels with diameters below 100 nm are called nanogels. Hence, increased amounts of APMH lead to the formation of nanogels, which are most-likely more useful for delivery of nucleic acids. Additionally, a stabilizing effect of these charged soluble polymers on the growing particles is possible, resulting in a further stabilization of small particles similar to the effect of surfactants [36,49]. This behavior differs from the behavior found for anionic copolymer microgels made with acrylic acid as comonomer. With increasing feed of an anionic monomer an increase of microgel dimensions is reported in the literature [59]. At the moment we can only speculate about the reasons for this inverse behavior. One explanation might be the already mentioned stabilization during nucleation. Another reason might be a different reaction pH.

The polydispersity index (PDI) obtained from the second cumulant, also shows a dependency on the APMH-feed. While the microgels with 0 or 2.5 mol% APMH exhibit a common polydispersity below 5%, microgels with 5 and 10 mol% APMH show increased values of (9.1±2.8) % and (24.9±3.5) %, respectively.

**Figure 2:**
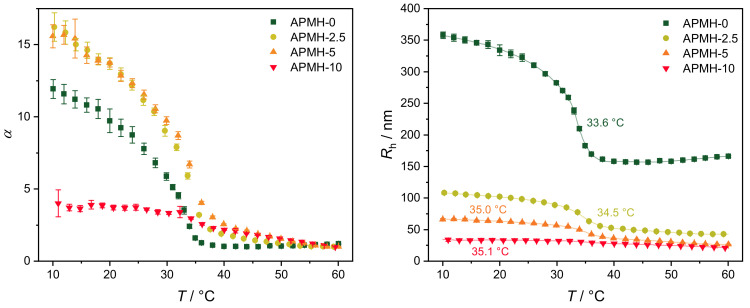
Temperature-dependent swelling behavior of the synthesized particles, shown by means of the hydrodynamic radius $R_{h}$ and the swelling ratio α. The corresponding VPTTs, determined from the point of inflection of cubic B-splines, are given in the right graph.

The temperature-dependent swelling behavior is shown in Figure 2 by means of the hydrodynamic radius $R_{h}$ and swelling ratio $\alpha ={\left(\frac{R_{h}\left(T\right)}{R_{h},\mathrm{collapsed}}\right)}^{3}$. While microgels with APMH feeds of 5 mol% or lower show a pronounced temperature induced collapse with initial swelling ratios between 10 and 17, the microgel APMH-10 only shows a minor temperature response. The corresponding volume phase transition temperatures (VPTT) increase by copolymerization of APMH from 33.6 ${}^{\circ }C$ to 35.1 ${}^{\circ }C$, indicating the successful incorporation of APMH into the microgel. A small apparent increase in $R_{h}$ for APMH-0 is observed for temperatures above 46 ${}^{\circ }C$, which is most-likely caused by increased attractive interaction between the microgels due to a decreased colloidal stability by a lack of stabilizing charges in combination with a large microgel size. As the copolymerization of APMH introduces additional charges, microgels with APMH are better stabilized at higher temperatures. In future works, a precise determination of microgel charges by measurements of the electrophoretic mobility is envisaged. Moreover, such experiments are also planed for the study of complexes of e.g., DNA and the cationic microgels [60].

**Figure 3:**
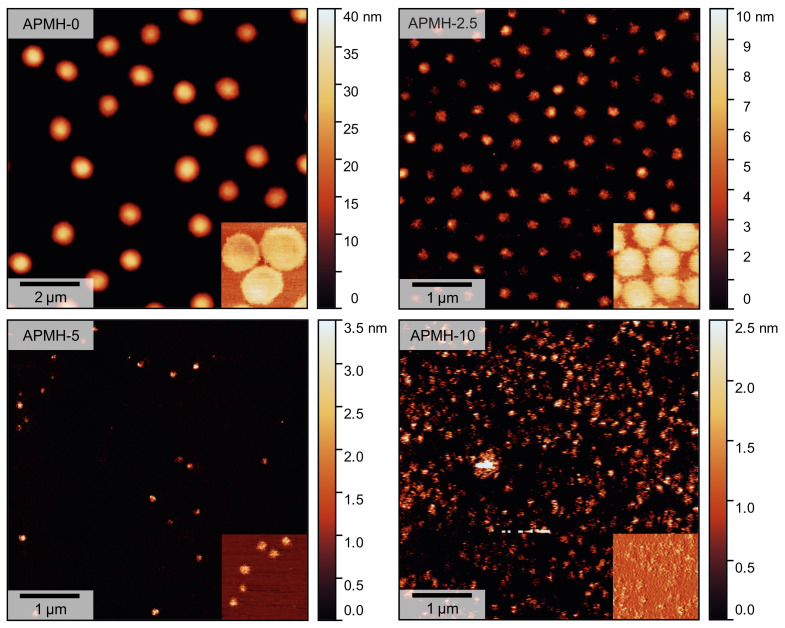
AFM images (topography) of the obtained poly(NIPAM-*co*-APMH) microgels. The insets show representative cutouts of the respective phase-image (same lateral scale). The samples were measured in the dried state at room temperature.

**Figure 4:**
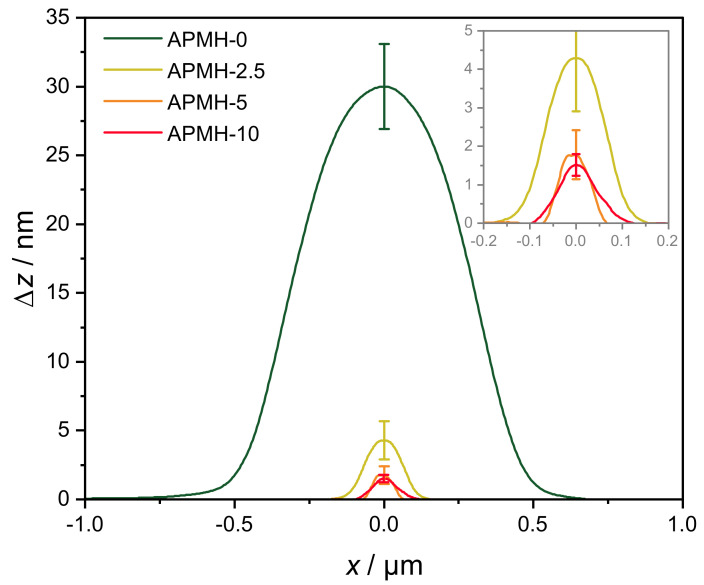
Average height profiles of poly(NIPAM-*co*-APMH) microgels extracted from topografic AFM images (Figure 3).

To analyze the influence of the cationic APMH on the size, shape and physical appearance of the microgels, atomic force microscopy (AFM) was performed on the samples in the dried state at room temperature. In Figure 3 both topographic height-images and phase-images are shown. The APMH-free microgels (APMH-0) can be recognized as particles with an uniform spherical appearance and low visual dispersity. Phase images of this microgel show a minor *core-corona* structure pointing to a highly cross-linked microgel core surrounded by a loosely cross-linked polymer corona and dangling polymer chains that are too thin to be seen in the height-image. Matching with PCS results, a decrease in size of the microgels can be seen with an increasing APMH-feed. Interestingly, the cross-section and appearance of the microgels become less circular and uniform compared to the APMH-0 particles. With the addition of 10 mol% APMH, it is difficult to find single particles or similarities in their shape, raising the question whether these particles can still be considered to be nano- or microgels or not. The chain collapse during synthesis is impeded by the cationic charges. Hence, irregularities in shape and internal network structure of the microgels can be expected. The averaged height of the microgels after deposition on the Si-wafers can be seen in Figure 4.

While APMH-free microgels exhibit a height of 30 nm, the copolymerization of APMH leads to very flat deposited microgels with heights of 5 nm and below.

Compared to the APMH-induced reduction in the hydrodynamic radius of the microgel, the reduction in height is more pronounced.

**Figure 5:**
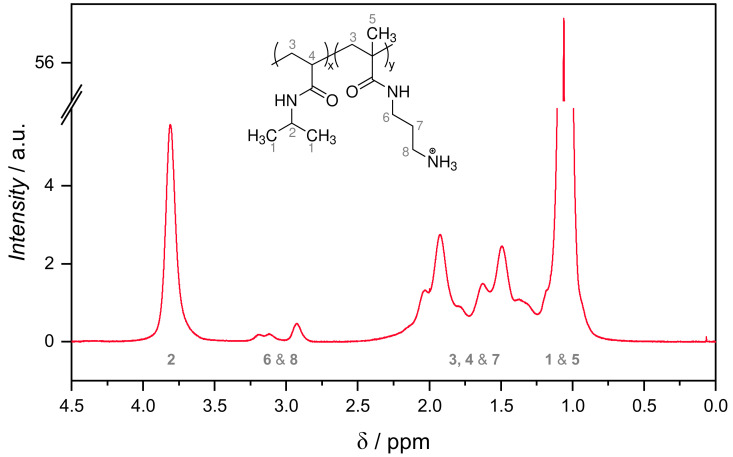
${}^{1}H-\mathrm{NMR}$ spectrum of the APMH-10 microgel in H_2_O/D_2_O. The assignment of signals was done according to [42]. Numbers below the peaks refer to the respective positions in the given structural formula. All other spectra are given in the Supplementary Materials.

For verification and quantification of APMH-incorporation into the microgel network, ${}^{1}H-\mathrm{NMR}$ spectra were recorded. As not all microgels were fully soluble in D_2_O after (freeze)drying, all ${}^{1}H-\mathrm{NMR}$ spectra were measured without drying from H_2_O/D_2_O-dispersions with 0.1 M DCl. The latter was added to ensure that all APMH units have the same protonation state (R−NH_3_^+^). A representative ${}^{1}H-\mathrm{NMR}$ spectrum of microgel APMH-10 is shown in Figure 5 with an assignment of signals to chemical structure.

The presence of signals 6 and 8 in the vicinity of 3 ppm is a proof of APMH incorporation into the microgel network. For the APMH-free microgel APMH-0, no signals are found in the range of 2.2–3.6 ppm. This confirms the correct assignment of signal 6 and 8 to APMH and ensures that no other signals (BIS, V50) are present in this interval. For the quantification of incorporated APMH, the integral ratio of signal 2 (NIPAM, 1H) and both 6 and 8 (APMH, 4H) were used. The incorporated APMH amount and its relation to the APMH-feed (incorporation success) are listed in Table 2. The amount of APMH inside the microgel network increases as expected with the APMH-feed. However, only less than one third of APMH is built-in into the microgels due to the formation of APMH-rich soluble polymers that are not incorporated into the microgels. It has to be noted, that the absolute error of APMH determination by ${}^{1}H-\mathrm{NMR}$ is elevated due to a low signal-to-noise ratio and the necessary baseline correction.

### 3.2. Variation of Reaction pH

Like discussed above, the incorporation of the cationic APMH leads to several unwanted side-effects due to the cationic charges being present during microgel synthesis. For possible future applications, microgels with better yields, lower polydispersities and more effective APMH-incorporation are desirable. As APMH (and most other similar monomers) are sold as hydrochlorides with an excess of hydrochloric acid, the reaction pH without adjustment is very acidic (down to 2.6 in our case). Hence, the APMH is protonated entirely. With the $pK_{a}$ of approximately 10, first effects on microgel synthesis should be visible for a reaction pH higher than 8 or 9 [36]. To investigate the influence of the reaction pH on synthesis, microgels with 10 mol% APMH were synthesized at various reaction pH values between 2.6–10.5. In this study, the reaction pH was simply adjusted by adding small amounts of sodium hydroxide solution and hydrochloric acid to the heated reaction mixture prior to initiation. Therefore, the additional ionic strength is kept as low as possible. As with this method the reaction mixture is not buffered, a change in pH during the course of reaction might occur, leading to a compositional drift. Despite this problem, the use of buffers is not ideal, as they need a rather high ionic strength to work properly and different buffer systems are required to cover a large pH interval.

With rising the reaction pH up to 9.5, stable microgel suspensions with increasing turbidity were obtained. An overview on the performed syntheses is given in Table 3. Synthesis at pH = 10.0 and 10.5 is considered unsuccessful with 0% microgel yield, as large amounts of macroscopic gels were formed after initiation (see Figure S1). Apart from this limitation, the microgel yield could be doubled by the adjustment of reaction pH, where the most distinct increase in yield is seen for pH = 9.0 and 9.5, where the pH approaches the $pK_{a}$ of APMH. As a raised reaction pH increases the total amount of deprotonated (uncharged) APMH, the impact on the chain-collapse is attenuated. Therefore, more polymeric material is incorporated into the microgels and does not remain as water-soluble polymer, which is confirmed by the decrease of WSP from 73% (pH = 2.6) to 35% (pH = 9.5). Yield (and WSP) at a reaction pH of 2.6 are nearly as high (low) as with the APMH-free synthesis APMH-0 (Table 1), demonstrating a good improvement of microgel yield through pH-adjustment.

**Figure 6:**
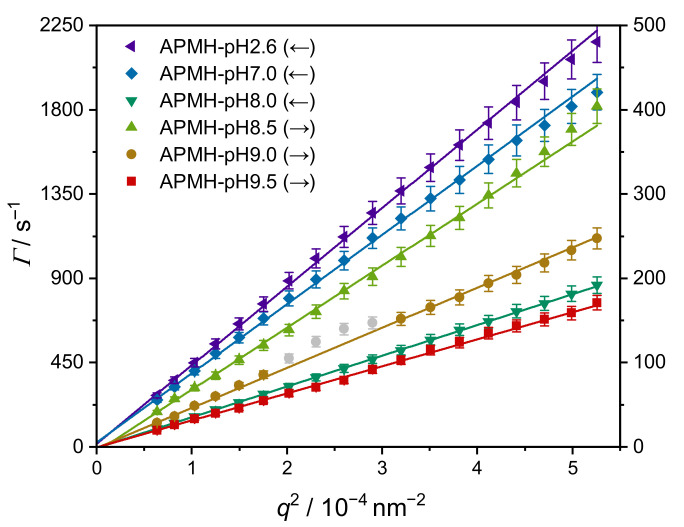
Relaxation rates Γ measured by angle-dependent PCS plotted against $q^{2}$ with linear regressions. Grey data points were excluded due to deviations resulting from a formfactor minimum. The corresponding ordinate is indicated by an arrow in the legend.

The hydrodynamic radii $R_{h}$ and PDI at 10 ${}^{\circ }C$ were obtained by angle-dependent PCS measurements and are given in Table 3. The corresponding plots of Γ against $q^{2}$ are shown in Figure 6 with the expected linear dependence. Deviations due to a local formfactor minimum in combination with an elevated polydispersity are present for the microgel APMH-pH9.0. The influence of reaction pH on the hydrodynamic radius is strong, as microgel APMH-pH9.5 ($R_{h}=(493\pm 25)\mathrm{nm}$) is 13-fold larger than microgel APMH-pH2.6 without pH-adjustment. Analogous to the increase in yield, the suppression of cationic charges promotes the necessary chain-collapse during synthesis leading to a lower amount of water-soluble polymer. With more material being incorporated into the microgels, their size logically increases.

**Figure 7:**
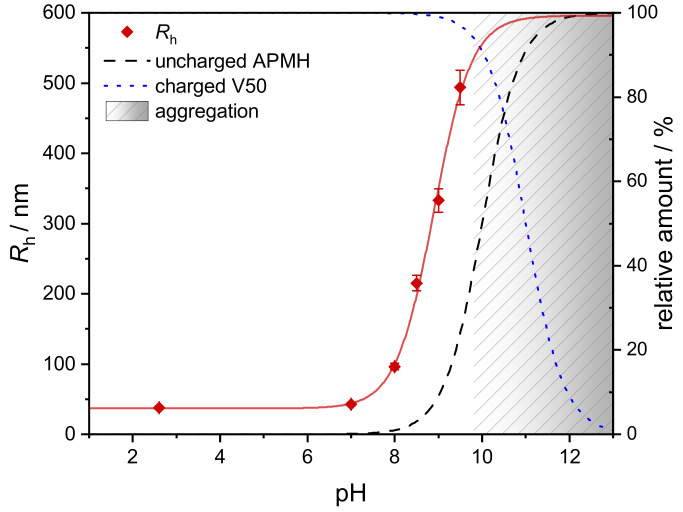
Dependence of $R_{h}$ on the reaction pH with a modified Henderson-Hasselbalch adjustment after Equation (4). Additionally shown are the protonation states of APMH and V50 for $pK_{a}$-values of 10 and 11 [61], respectively. The region above pH = 9.5 is not accessible due to aggregation during synthesis.

When plotting $R_{h}$ against the reaction pH (Figure 7), a steep increase is seen for pH>8. More interesting, the data is reminiscent of a Henderson-Hasselbalch function as it runs parallel with the relative amount of uncharged APMH monomers. Since a direct dependence of microgel size and protonation state of the APMH can be assumed, the hydrodynamic radii were fitted with a modified Henderson-Hasselbalch function (4), consisting of a maximum and minimum value for $R_{h}$ and the inflection point β.

(4) $$R_{h}\left(\mathrm{pH}\right)=\frac{10^{-\beta }}{10^{-\mathrm{pH}}+10^{-\beta }}\left(R_{h}^{max}-R_{h}^{min}\right)+R_{h}^{min}$$

In this case the parameters were obtained as β=8.895±0.074, $R_{h}^{max}=(596\pm 51)\;nm$ and $R_{h}^{min}=(36.8\pm 1.9)\;nm$. The inflection point β can be interpreted as an apparent $pK_{a}$ value and is lower than the real value of 10, given for APMH in the literature [36]. While the $pK_{a}$ value changes with temperature and usually shifts towards 7 upon polymerization, a lower value is not surprising. While the minimum radius is supported precisely by two microgels, the maximum value can only be estimated roughly due to the lack of data above pH = 9.5. The latter is most-likely induced by the starting deprotonation of the initiator V50 together with an increased ionic strength and large microgel size, leading to an insufficient stabilization of microgels at elevated temperature. The tendency to aggregate is also observable for the purified microgels, when they are heated under basic conditions (see Figure S2). Other than the aggregation during synthesis, this phenomenon is reversible as no covalent bonds are formed. The aggregation of similar microgels at pH = 12 was reported by Gelissen et al. for temperatures above 40 ${}^{\circ }C$ by PCS measurements [62].

Besides the change in reaction pH, the resulting increase in ionic strength has to be discussed. As mentioned before, the reaction pH was adjusted with small amounts of acid/base. Therefore, the ionic strength increases together with reaction pH. Known from literature, an elevation of ionic strength by addition of NaCl leads to larger microgels when APMH is copolymerized due to a charge-screening effect [12,36]. Seen from Figure 7, the change in $R_{h}$ is very low for pH≤8 and negligible for pH≤7, while the strongest change in $R_{h}$ is seen for pH>8. Additionally, the synthesis APMH-pH9.5 was repeated, but instead of NaOH, the same molar amount of NaCl was added. This way, a synthesis at very acidic pH (2.7) but with similar additional ionic strength as in APMH-pH9.5 was performed, leading to microgels with $R_{h}=(40.0\pm 2.0)\;nm$. Therefore, a clear influence of the change in ionic strength by pH adjustments can be neglected. The deprotonation of APMH by the elevated reaction pH can be considered to be the main reason for the observed effects.

Concerning the polydispersities of the microgels (see Table 3), a reduction in PDI is seen for reaction pH of 8.0 and above. While PDI of APMH-pH7.0 ((32.7±3.1) %) is raised compared to initial synthesis APMH-pH2.6 ((24.9±3.5) %), the lowest PDI is observed for APMH-pH9.0 ((4.4±2.8) %) and is similar to the PDI of the APMH-free synthesis (APMH-0). The small increase of PDI at pH 9.5 might be a forecast of the aggregation occurring at higher pH.

**Figure 8:**
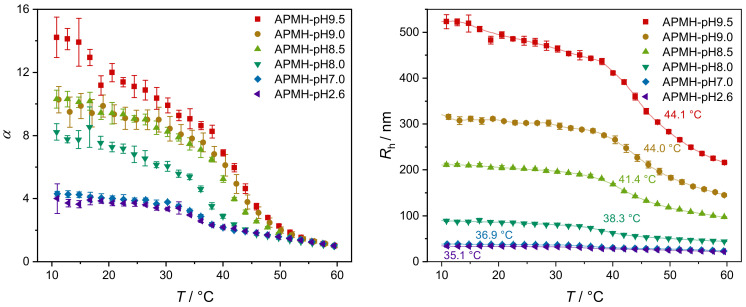
Temperature-dependent swelling behavior, plotted by means of the hydrodynamic radius $R_{h}$ and the volume swelling ratio α. The corresponding VPTTs, determined from the point of inflection of the cubic B-splines, are given in the right graph.

The influence of the reaction pH on the temperature-induced swelling behavior is shown in Figure 8. Besides the differences in $R_{h}$, a significant broadening and shift in the volume phase transition can be seen with increasing reaction pH. By adjusting the pH to 9.5, the VPTT increased by 9 ${}^{\circ }C$ from 35.1 ${}^{\circ }C$ (APMH-pH2.6) to 44.1 ${}^{\circ }C$, indicating an increasing content of APMH inside the microgel network. The shift and broadening of the VPT are so pronounced that a full collapse of the microgels is not visible in the examined temperature range. Despite the restrained collapse with increasing reaction pH, the swelling ratio α increases simultaneously from around 4 (APMH-pH2.6 and -pH7.0) up to 14 (APMH-pH9.5).

**Figure 9:**
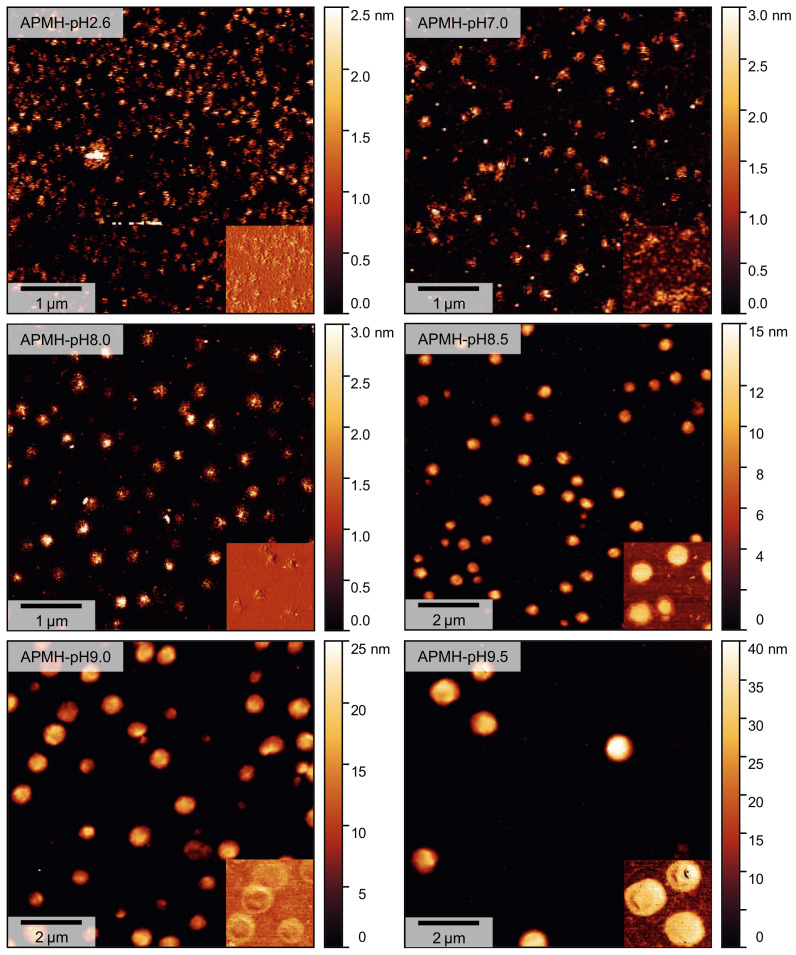
AFM images (topography) of the obtained poly(NIPAM-*co*-APMH) microgels, synthesized at different reaction pH. The insets show representative cutouts of the respective phase-image (same lateral scale). The samples were measured in the dried state at room temperature.

The influence of the reaction pH on size, shape and physical appearance of the microgels was again analyzed by atomic force microscopy in tapping-mode. The topographic images including cutouts from the respective phase images are shown in Figure 9. In agreement with the PCS data, the increase in microgel size with a more basic reaction pH is visible. Again, microgels synthesized at low pH appear to be less spherical and more fuzzy. For higher reaction pH the microgels have larger dimensions and are clearly discernible as single particles with a circular appearance, compatible with the expected spherical shape in bulk solution. As the amount of charged APMH is reduced with increasing reaction pH, the formation of larger microgels with a lower dispersity was expected. Compared to the APMH free microgel APMH-0, these microgels are visibly more disperse in their size, though. This increase in apparent polydispersity might arise from the initiation kinetics, since V50 is also deprotonateable at high pH, but its exact origin remains unclear and gives possibility for further improvement.

**Figure 10:**
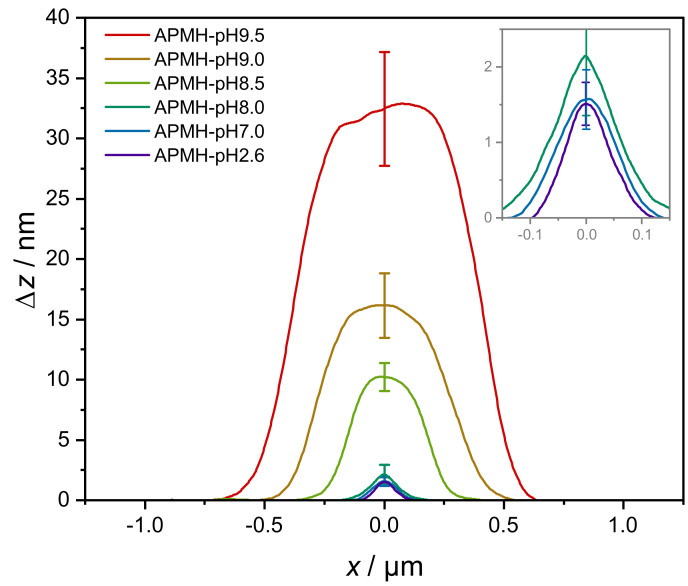
Average height profiles of poly(NIPAM-*co*-APMH) microgels, synthesized at different pH, extracted from Figure 9.

Consistent with PCS data, the microgel height profiles (Figure 10) show a strong increase in height with a more basic reaction pH. The most prominent change in diameter and height occurs for reaction pH over 8.0, where the quantitative deprotonation of APMH is starting (see Figure 7).

**Figure 11:**
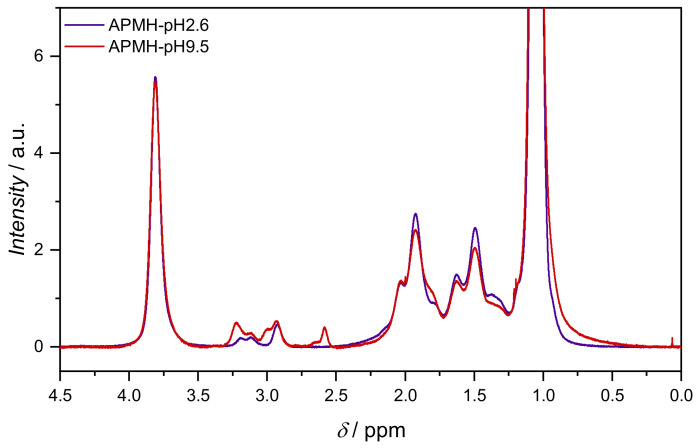
${}^{1}H-\mathrm{NMR}$ spectra of microgel APMH-pH2.6 and -pH9.5 in H_2_O/D_2_O. With increasing pH, a change in APMH-signals is visible. All spectra are given in the Supplementary Materials.

The effect of reaction pH on APMH-incorporation was again monitored with ${}^{1}H-\mathrm{NMR}$ spectroscopy. Comparing spectra of APMH-pH2.6/APMH-0 with APMH-pH9.5, a change in the APMH-signals is visible (Figure 11). The new NMR signals indicate that the APMH moieties are only partially protonated, leading to different chemical shifts. Despite the very acidic measurement conditions, moieties deep inside the microgel could be deprotonated nevertheless. As a change in microgel network structure by the increased APMH-incorporation is suspected, the interaction of neighboring APMH moieties by hydrogen bonding is possible, too. APMH incorporation was determined as before, with the APMH signals (2.4–3.4 ppm) evaluated as four protons. The obtained values are listed in Table 4.

As presumed from the increasing VPTT (see Figure 8), the general enhancement of APMH-incorporation by elevation of reaction pH is confirmed by ${}^{1}H-\mathrm{NMR}$ measurements. With rising the reaction pH from initially 2.6 up to 9.5, the APMH-incorporation more than doubled with a maximum incorporation success of 75%. As aggregation occurs for reaction pH above 9.5, a quantitative incorporation of APMH was not achieved.

## 4. Conclusions

A precise pH-control during the synthesis of pH-responsive microgels is important for reproducibility and comparability of the results. From the variation of the APMH-feed in poly(NIPAM-*co*-APMH) microgels, a negative influence of increasing molar ratios of the comonomer on dispersity and yield was found. The addition of APMH also led to a strong decrease in microgel size, while the overall APMH incorporation was below one third, compared to the APMH-feed. For a nominal feed of 10 mol% at pH = 2.6 nanogels were obtained. These effects are traced back to the APMH being charged during reaction. By elevating the reaction pH up to 9.5, the microgel yield, size and APMH-incorporation increased. The dispersity could also be decreased, but is still elevated in comparison with APMH-free microgels.

Hence, the reaction pH could be used as a parameter to improve the synthesis of poly(NIPAM-*co*-APMH) microgels or to tailor their size in surfactant free syntheses. As the effects are caused by the protonation state of the APMH, the results should also be applicable to other (de)protonateable comonomers.

More generally and importantly, the impact of the reaction pH on microgel synthesis should be kept in mind when synthesizing microgels containing pH-responsive moieties. For reproducible results, the reaction pH has to be kept constant or at least far away from the respective $pK_{a}$-value of the pH-dependent monomers.

## Figures and Tables

**Table 1:** Overview of the synthesized poly(NIPAM-*co*-APMH) microgels with different amounts of APMH (feed), with respect to the molar amount of NIPAM. Additionally, the reaction pH prior to initiation, hydrodynamic radii ($R_{h}$) and polydispersities (PDI) at 10 ${}^{\circ }C$, microgel yield and amount of water soluble polymer (WSP) in the supernatant of the first centrifugation step are shown.

Microgel	APMH/mol%	pH	$R_{h}$ (10 °C)/nm	PDI/%	Yield/%	WSP/%
APMH-0	0.0	7.6	361 ± 18	4.6 ± 3.9	54	32
APMH-2.5	2.5	3.3	110.7 ± 5.5	4.6 ± 4.1	41	37
APMH-5	5.0	3.1	68.2 ± 3.4	9.1 ± 2.8	39	45
APMH-10	10.0	2.6	37.7 ± 1.9	24.9 ± 3.5	24	73

**Table 2:** APMH content in the microgels relative to the amount of NIPAM, measured by ${}^{1}H-\mathrm{NMR}$ spectroscopy.

Microgel	APMH Content/%	Incorporation Success/%
APMH-0	0.0	-
APMH-2.5	0.6	24
APMH-5	1.2	24
APMH-10	3.1	31

**Table 3:** Overview on synthesized poly(NIPAM-*co*-APMH) microgels with 10 mol% APMH-feed, synthesized at different reaction pH. Additionally, the hydrodynamic radii and dispersity (PDI) at 10 ${}^{\circ }C$, microgel yield and amount of water-soluble polymer (WSP) in the first supernatant of centrifugation are shown.

Microgel	APMH/mol%	pH	$R_{h}$ (10 °C)/nm	PDI/%	yield/%	WSP/%
APMH-pH2.6 ^1^	10.0	2.6 ^2^	37.7 ± 1.9	24.9 ± 3.5	24	73
APMH-pH7.0	10.0	7.0	42.8 ± 2.1	32.7 ± 3.1	28	63
APMH-pH8.0	10.0	8.0	96.4 ± 4.8	7.0 ± 2.8	29	50
APMH-pH8.5	10.0	8.5	215 ± 1.1	5.6 ± 3.3	28	47
APMH-pH9.0	10.0	9.0	333 ± 17	4.4 ± 2.8	41	38
APMH-pH9.5	10.0	9.5	493 ± 25	8.3 ± 4.5	47	35
APMH-pH10.0 ^3^	10.0	10.0	-	-	0	-
APMH-pH10.5 ^3^	10.0	10.5	-	-	0	-

^1^ Note: The name APMH-pH2.6 references the same microgel as APMH-10 (see Table 1) does. ^2^ This is the intrinsic pH without further adjustment. ^3^ Macroscopic aggregation of polymeric material occurred shortly after initiation.

**Table 4:** APMH content in the microgels relative to the amount of NIPAM, measured by ${}^{1}H-\mathrm{NMR}$ spectroscopy. The nominal APMH-feed was 10 mol% in all cases.

Microgel	APMH Content/%
APMH-pH2.6	3.1
APMH-pH7.0	3.2
APMH-pH8.0	6.1
APMH-pH8.5	6.4
APMH-pH9.0	7.5
APMH-pH9.5	7.4

## Data Availability

The data presented in this study are available on request from the corresponding author.

## Supplementary Materials

The following are available online at https://www.mdpi.com/2073-4360/13/5/827/s1, Figure S1: Aggregation during synthesis, Figure S2: Stability of microgels regarding pH and temperature, Figures S3–S11: ${}^{1}H-\mathrm{NMR}$ spectra.
