# Supplementary material for: Importance of pH in Synthesis of pH-Responsive Cationic Nano- and Microgels
Source: Polymers (Basel). 2021 Mar 8;13(5):827. doi: 10.3390/polym13050827 (PMC7962641; doi:10.3390/polym13050827)
Supplement: Supplementary file 1 [file polymers-13-00827-s001.pdf]

Supplementary Materials: Importance of pH in Synthesis of pH-responsive Cationic Nano- and Microgels

Marco Annegarn [1], Maxim Dirksen [1] and Thomas Hellweg [1,*]

1. Aggregation during Synthesis

The synthesis of poly(NIPAM-*co*-APMH) microgels was only possible for a reaction pH of 9.5 or below. Aggregation occurred for more basic conditions, impeding the successful synthesis of microgels (Fig. S1).

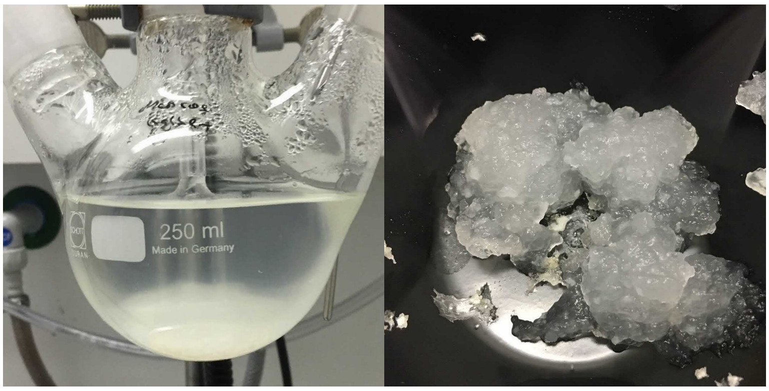

Figure S1. For synthesis at pH 10 and 10.5, the formation of visible aggregates occurred shortly after initiation. The aggregation is likely caused by the decreased amount of surface charges and elevated ionic strength. The pictures show the water-swollen aggregates that can be characterized as macroscopic gels. The pictures refer to the syntheses APMH-pH10.5 (left) and APMH-pH10.0 (right).

2. Stability of Microgels Regarding pH and Temperature

For acidic and neutral conditions, the synthesized poly(NIPAM-*co*-APMH) microgels were stable even at elevated temperatures. For basic conditions, reversible aggregation occurs upon heating (Fig. S2).

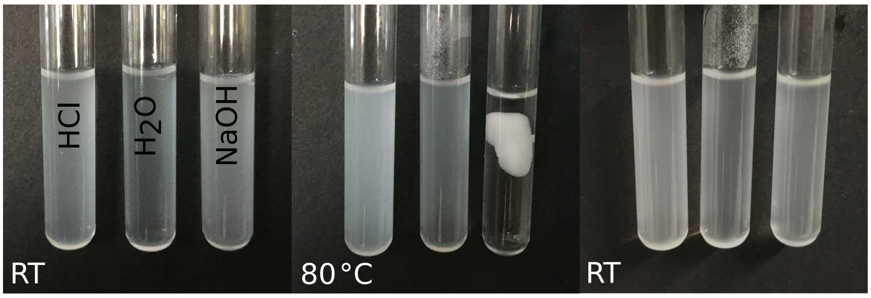

Figure S2. Demonstration of microgel stability with APMH-pH9.0. The microgels were dissolved in 0.05 M HCl, H_2O and 0.05 M NaOH, respectively. The pictures were taken at room temperature (RT), after heating in a drying oven (T≈80 °C) and after cooling back to RT and shaking. Macroscopic aggregation only occurs under basic conditions at elevated temperatures.

3. ^{1}H-NMR Spectra

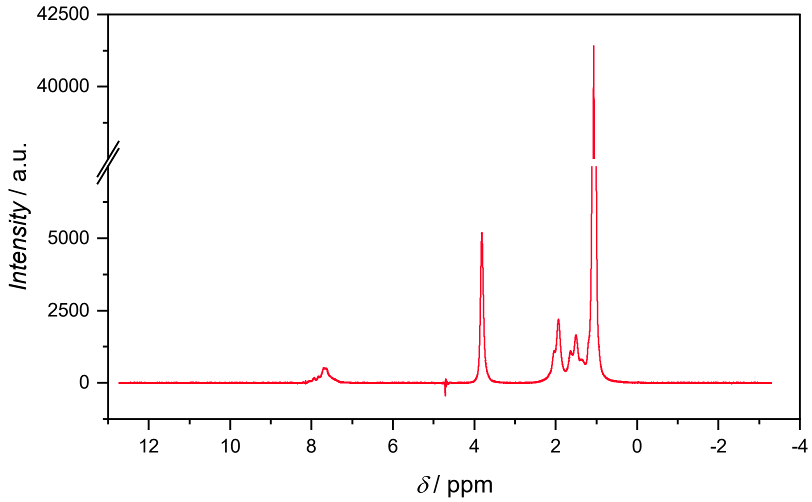

Figure S3. ^{1}H-NMR spectrum of microgel APMH-0.

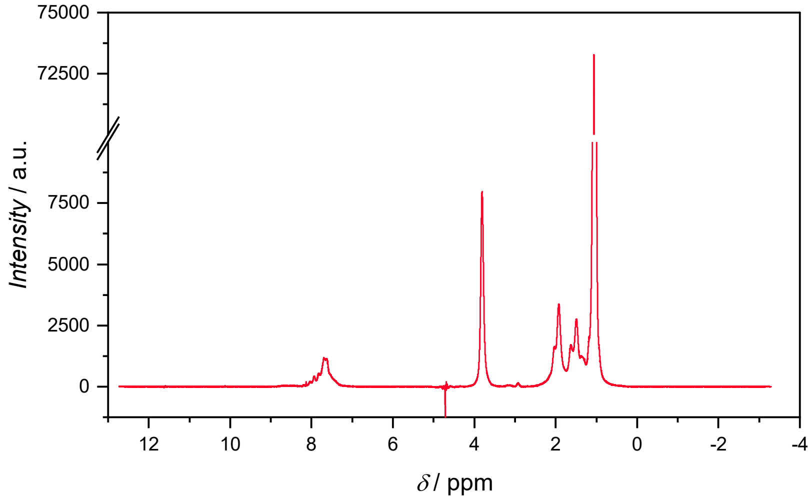

Figure S4. ^{1}H-NMR spectrum of microgel APMH-2.5.

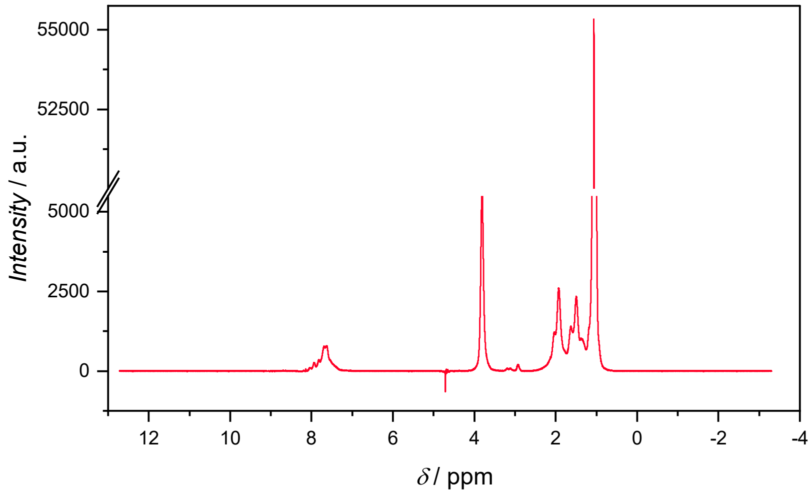

Figure S5. ^{1}H-NMR spectrum of microgel APMH-5.

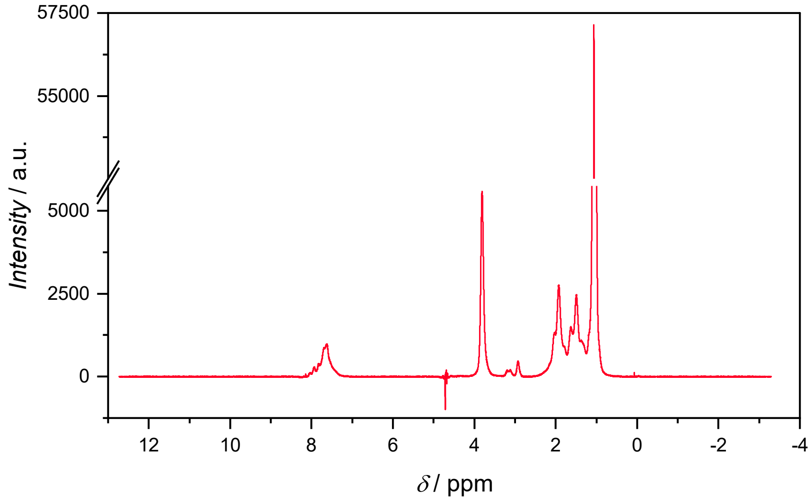

Figure S6. ^{1}H-NMR spectrum of microgel APMH-10/APMH-pH2.6.

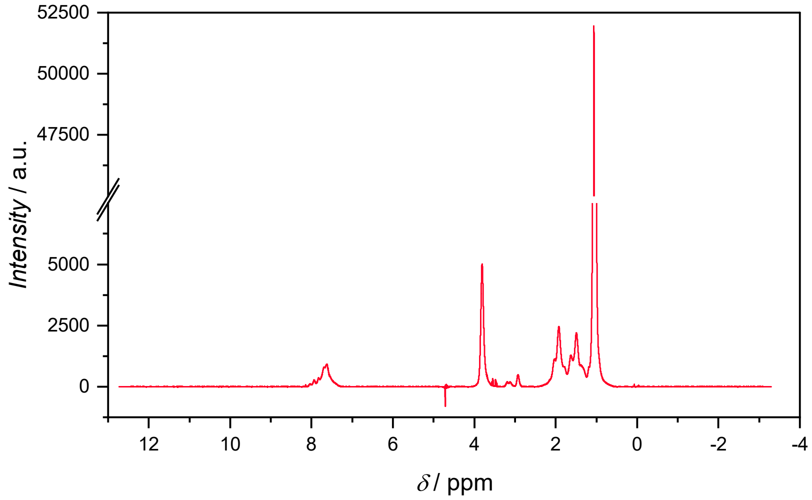

Figure S7. ^{1}H-NMR spectrum of microgel APMH-pH7.0.

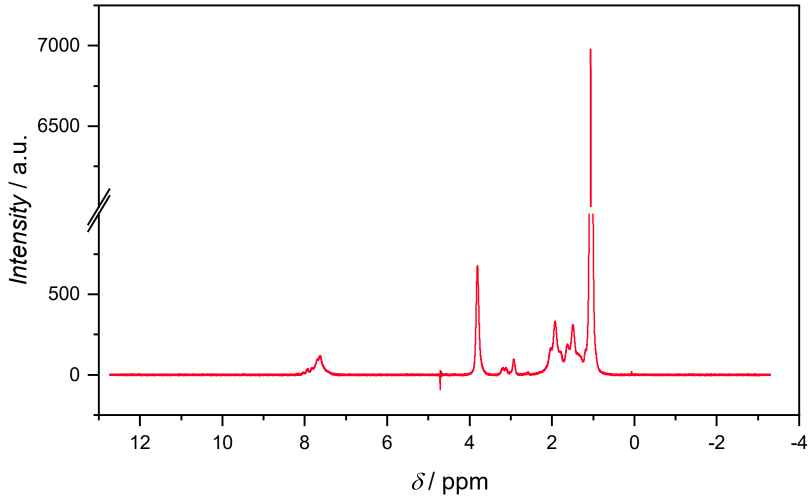

Figure S8. ^{1}H-NMR spectrum of microgel APMH-pH8.0.

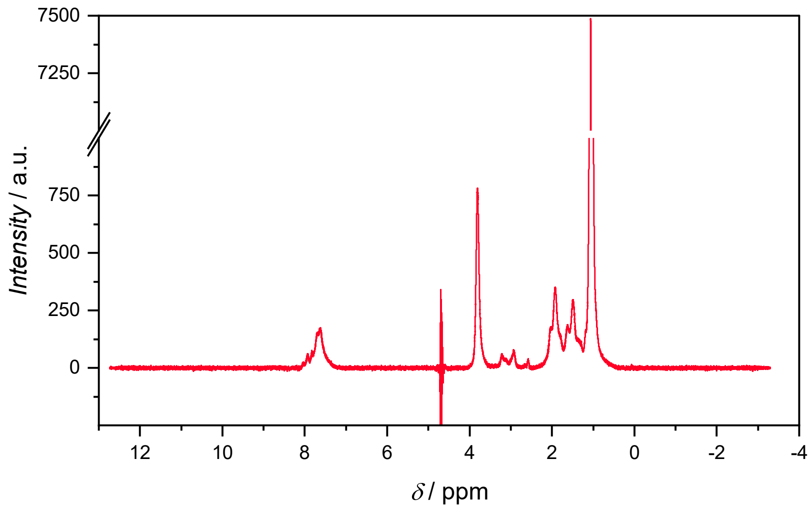

Figure S9. ^{1}H-NMR spectrum of microgel APMH-pH8.5.

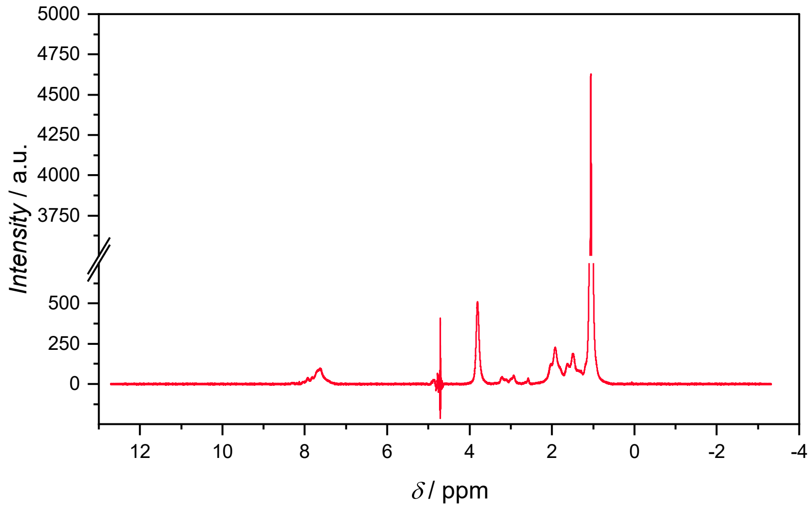

Figure S10. ^{1}H-NMR spectrum of microgel APMH-pH9.0.

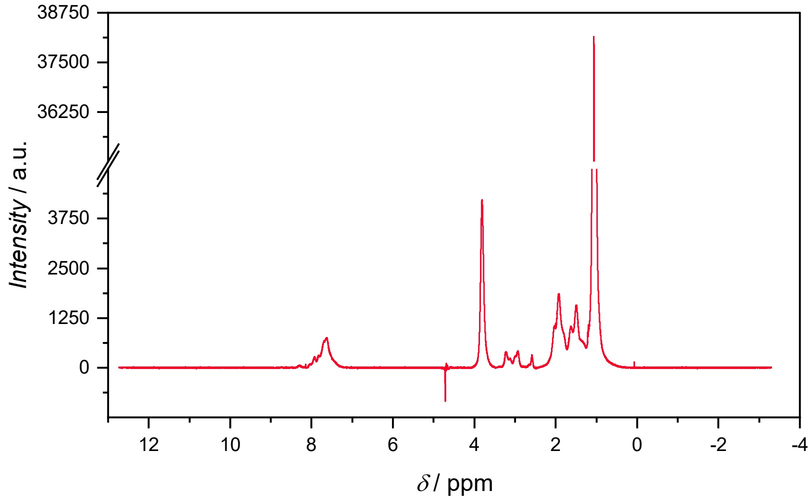

Figure S11. ^{1}H-NMR spectrum of microgel APMH-pH9.5.
